# Exploring the relationship between frailty and executive dysfunction: the role of frontal white matter hyperintensities

**DOI:** 10.3389/fnagi.2023.1196641

**Published:** 2023-08-30

**Authors:** Natalia Pozo, César Romero, Maricarmen Andrade, Paul H. Délano, Vicente Medel, Marco Troncoso, Patricia Orellana, Maria Isabel Rodriguez, Camila Fabres, Carolina Delgado

**Affiliations:** ^1^Department of Neurology, Hospital San Borja Arriarán, Santiago, Chile; ^2^Department of Neurology and Neurosurgery, Hospital Clínico de la Universidad de Chile, Santiago, Chile; ^3^Department of Geriatric Medicine, Clínica Universidad de los Andes, Santiago, Chile; ^4^Department of Neuroscience, Faculty of Medicine, Universidad de Chile, Santiago, Chile; ^5^Department of Otorhinolaryngology, Hospital Clínico de la Universidad de Chile, Santiago, Chile; ^6^Advanced Center for Electrical and Electronic Engineer (AC3E), Valparaíso, Chile; ^7^Latin American Brain Health Institute (BrainLat), Universidad Adolfo Ibáñez, Santiago, Chile; ^8^Department of Radiology, Hospital Clínico de la Universidad de Chile, Santiago, Chile; ^9^Geriatric Unit, Internal Medicine Service, Hospital Puerto Montt Dr. Eduardo Schütz Schroeder, Puerto Montt, Chile

**Keywords:** dementia, frailty, executive dysfunction, white matter hyperintensities, older adults, vascular cognitive impairment, subcortical

## Abstract

**Introduction:**

Frailty is a geriatric syndrome frequently associated with executive dysfunction and white matter hyperintensities (WMH). But the relation between executive dysfunction and brain changes is poorly understood in frail subjects. Our hypothesis is that frontal-WMH mediates the association between frailty and executive dysfunction.

**Methods:**

A convenience sample of 113 subjects older than 65 years without dementia was studied with neuropsychological test, a structured clinical interview, physical examination and brain MRI. They were classified as robust or pre-frail and frail using the frailty phenotype score (0–5). The frontal WMH (F-WMH) were manually graduated (0–6) using the “Age-Related White Matter Changes score” from FLAIR sequences at a 3 Tesla brain MRI. A mediation analysis was done for testing whether F-WMH could act as a link factor between frailty phenotype score and executive dysfunction.

**Results:**

The group’s mean age was 74 ± 6 years, subjects with higher frailty score had more depressive symptoms and worse performance in executive function tests. A regression analysis that explained 52% of the variability in executive functions, revealed a significant direct effect of frailty score (Standardized βcoeff [95% CI] −0.201, [−0.319, −0.049], and F-WMH (−0.152[−0.269, −0.009]) on executive functions, while the F-WMH showed a small partial mediation effect between frailty and executive functions (−0.0395, [−0.09, −0.004]).

**Discussion:**

Frontal matter hyperintensities had a small mediation effect on the association between frailty and executive dysfunction, suggesting that other neuropathological and neurofunctional changes might also be associated with executive dysfunction in frail subjects.

## 1. Introduction

Frailty is a clinical syndrome of decreased functional reserve that occurs as a result of physiological decline in multiple systems that is different from normal aging (Clegg et al., 2013). It manifests as a state of vulnerability associated with greater risk for adverse health-related outcomes, including falls, disability, dementia, institutionalization and death (Fried et al., 2001; Clegg et al., 2013).

The prevalence of frailty varies significantly according to the population studied and the tools used, with an average estimated prevalence of 4–16% in adults 65 years and older in the United States (Fried et al., 2001; Bandeen-Roche et al., 2006; Cawthon et al., 2007; Kiely et al., 2009). In Latin America and the Caribbean, the mean prevalence of frailty in adults 60 years and older is 19.6% (ranging from 7.7 to 42.6%) (Da Mata et al., 2016).

Operationally, physical frailty is defined by the frailty phenotype criteria as presence of at least 3 out of 5 of the following signs or symptoms: weight loss, exhaustion, slowness, low physical activity and weakness (Fried et al., 2001). Another way to define frailty is based on a multidimensional frailty index, which considers an accumulation of health conditions associated with aging (Rockwood et al., 2005).

Frailty syndrome has been associated with global cognitive impairment, especially with executive dysfunction and lower processing speed (Robinson et al., 2022). When frailty is associated with mild cognitive impairment the term “cognitive frailty” is used (Kelaiditi et al., 2013). The neuropsychological profile of people with cognitive frailty is characterized primarily by executive dysfunction and attentional deficits, which in addition had slower gait speed, likely to a subcortical frontal syndrome (Delrieu et al., 2016). Although physical frailty has been proposed as a significant predictor of Alzheimer’s disease (Clegg et al., 2013), it has been particularly related also with vascular and non-Alzheimer’s disease dementia (Borges et al., 2019).

Interestingly, evidence shows that people with cognitive frailty have larger brain regions with indicators of ischemic vascular disease, such as white matter hyperintensities (WMH) and lacunes (Sugimoto et al., 2019; Yoshiura et al., 2022). In fact, WMH are also associated with executive dysfunction and lower processing speed (Buchman et al., 2013; Prins and Scheltens, 2015), as well as slower gait and reduced mobility (Zheng et al., 2011). Thus, possibly vascular mechanisms are implicated in cognitive impairment and lower gait in frail patients, as it had been suggested by longitudinal studies that had shown that higher brain volumes of WMH are associated with frailty progression over time (Maltais et al., 2019; Siejka et al., 2020; Ducca et al., 2023).

Considering that the subcortical frontal cognitive profile is a common feature between subjects with cognitive frailty and those with extensive WMH, and that cognitive frail subjects have larger WMH, we hypothesized that subcortical WMH in the frontal areas mediate the association between frailty and executive dysfunction. The objective of this study is to unravel whether subcortical WMH mediates the association between frailty and executive dysfunction in independent older adults without dementia.

## 2. Methods

### 2.1. Participants

A total of 142 patients aged 65 years and older from primary care public health centers from the Recoleta area of Santiago de Chile were screened between 2016 and 2018. Participants were selected according to the inclusion and exclusion criteria from the Auditory and Dementia study (ANDES) which aims to study the associations between hearing loss and cognitive impairment (Belkhiria et al., 2019). Inclusion criteria were (i) age 65 years and older at the beginning of the study, (ii) to have a Mini-Mental State Examination (MMSE) score of ≥24, and (iii) demonstrate preserved functionality measured by a Pfeffer’s Functional Activities Questionnaire score <2 (Quiroga et al., 2004). Exclusion criteria were (i) having a stroke or other symptoms of neurological disorders, (ii) having dementia, (iii) prior psychiatric disorders, (iv) displaying other causes of hearing loss different from presbycusis, (v) using hearing aids, and (vi) other causes of significant disability, such as poor vision (Snellen test ≥ 50/20) or severe arthrosis. Participants provided written informed consent. The study adhered to ethical guidelines of the Declaration of Helsinki (1996).

### 2.2. Subject evaluations

#### 2.2.1. Clinical assessment

Patients were evaluated with a structured interview about biomedical conditions and physical examination. The Charlson comorbidity index was calculated to weigh the burden of comorbid disease (Charlson et al., 1987).

Frailty score was calculated using the following Cardiovascular Health Study criteria, according to the presence (score = 1) or absence (score = 0) of: 1.- self-reported unintentional weight loss of more than 10 pounds in the past year, 2.- self-reported decrease in energy in the past year, 3.- muscle weakness measured by a handgrip strength <33 pounds in women and <59 pounds in men, 4.- slow gait speed in a 4, 5 meters distance (>6 s), and 5.-self-reported low physical activity (Fried et al., 2001).

#### 2.2.2. Neuropsychological assessment

All subjects were evaluated with a structured interview and graded according to their cognitive complaints using the clinical dementia rating scale (CDRS) (Morris, 1993). Cognitive tests included the Mini-Mental State Examination (MMSE) for global cognition (Folstein et al., 1975), the Frontal Assessment Battery (FAB) (Dubois et al., 2000), and the Digit symbol test for measuring executive functions and the total recall of the Free and Cued Selective Reminding Test (FCSRT) to explore verbal episodic memory (Grober et al., 1988). Depressive symptoms were assessed by the examiner using the structured clinical interview for depression (SCID) of DSM-IV criteria, which assesses 10 clinical symptoms of depression (Gigantesco and Morosini, 2008).

#### 2.2.3. Audiological evaluations

Air and bone conduction audiometric thresholds were measured at octave frequencies from 125 to 8,000 Hz for each ear separately (AC40, Interacoustics^®^) by an experienced audiologist in a soundproof room placed in the Otolaryngology Department of the University of Chile Clinical Hospital. The air conducted thresholds at 500, 1,000, 2,000, and 4,000 Hz were averaged to calculate the pure-tone average (PTA) for each ear.

#### 2.2.4. Magnetic resonance imaging

Neuroimaging data were acquired with a MAGNETOM Skyra 3-Tesla on a magnetic resonance imaging (MRI) Scanner (Siemens Healthcare GmbH^®^, Erlangen, Germany) equipped with a head volume coil. T1-weighted magnetization-prepared rapid gradient echo (T1-MPRAGE) axial images were collected, and parameters were as follows: time repetition (TR) = 2300 ms, time echo (TE) = 232 ms, matrix = 256 × 256, flip angle = 8°, 26 slices, and voxel size = 0.94 mm × 0.94 mm × 0.9 mm. T2-weighted turbo spin echo (TSE) (4500 TR ms, 92 TE ms) and fluid attenuated inversion recovery (FLAIR) (8000 TR ms, 94 TE ms, 2500 TI ms) were also collected to inspect structural abnormalities. A total of 440 images were obtained during an acquisition time of 30 min per subject.

##### 2.2.4.1. White matter hyperintensities (WMH)

White matter hyperintensities were defined as areas with a brighter signal intensity than the surrounding white matter on fluid attenuated inversion recovery (FLAIR) on brain MRI (Tubi et al., 2020). They were manually rated by an experienced neuroradiologist from axial slices of FLAIR images by using the “Age-Related White Matter Changes score (ARWMC)” (Wahlund et al., 2001) which rates the WMH in 4 levels from 0 to 3 (no lesions, focal lesions, confluent lesions, and diffuse involvement of an area) in five areas bilaterally: frontal, parieto-occipital, temporal, basal ganglia, and infratentorial.

### 2.3. Data analysis

SPSS software version 23 was used to perform the statistical analysis. We explored demographic, clinical, neuropsychological, and neuroimaging data. We used descriptive statistics with mean and standard deviation for continuous variables, median (range) for ordinal variables and numbers and percentages for category variables. In line with newer studies that included pre-frail individuals at an increased risk of cognitive decline (Ruan et al., 2015) we analyzed the pre-frail and frail groups as one. Clinical, neuropsychological and neuroimaging data were compared between robust (Frailty phenotype score = 0) and pre-frail and frail subjects (scores >0). Given these two groups, we compared previous variables using ANCOVA, applying age, sex, and years of education as covariates. Because our main aim was to study the possible mediation effects of the WMH and executive functions, we focused only on bilateral frontal WMH (sum of the right and left scores of frontal ARWMC scores 0–6), for comparing the bilateral frontal-WMH between robust and pre-frail and frail groups we used the Mann Whitney *U*-test. For measuring the executive function, we used a composite score, that was the average value of the Z scores of the frontal assessment battery and the digit symbol test. First, we computed Spearman correlation analyses between the three variables of interest: executive function, frontal-WMH and frailty phenotype scores. Further on, we did a mediation analysis for obtaining the direct and indirect effects between frail scores (independent variable X), frontal-WMH (mediator) and executive functions (dependent variable Y) using the software Process macro for SPSS version 4.2 beta (Hayes, 2023). This software used ordinary least squares regression for doing the mediation analysis (model 4), using three regression models, the first used the frontal-WMH scores as the dependent variable (Y), while the frailty scores (X), age, sex, and education were used as independent variables, allowing to obtain the direct effect of Frailty score over the frontal-WMH. In the second model, the executive function Z scores was used as the dependent variable, while the frailty scores, frontal-WMH scores, age, sex, and education were used as independent variables, obtaining the direct effect of frailty scores, and F-WMH over executive functions. Finally, in the third model, the executive function Z scores was used as the dependent variable, while the frailty scores, age, sex, and education were used as independent variables. Using this data the software calculated the total, direct and indirect effects (mediated by the frontal-WMH scores) of frailty scores over executive functions using 5000 bootstrap samples.

## 3. Results

### 3.1. Demographic variables

A total of 113 out of 142 subjects were included after applying the inclusion and exclusion criteria. The mean age of the participants was 74 ± 6 years (mean ± SD), while average schooling was 9 ± 4 years of education. The sample included 62% of females, which were significantly younger (73.1 ± 5.6 years old) than men (75.2 ± 5.1) (*T* = 2.0, *p* = 0.04). Seventy-four subjects were classified as robust (66%) and 39 as frail/pre-frail (35%) (35 pre-frail and 4 frail).

### 3.2. Comparison between the frail/pre-frail and robust groups

There were non-significant differences according to frailty status (frail/pre-frail vs. robust) in demographic variables (Table 1). After adjusting by age and education, the frail/pre-frail group had higher comorbidities represented by a higher Charlson index (1.24 ± 2.04 vs. 0.67 ± 1.46; *p* = 0.02) and lower gait speed in the timed up and go test (9.49 ± 4.92 vs. 8.27 ± 3.53 s; *p* = 0.037) than the robust one. In the neuropsychological evaluation, the frail/pre-frail group performed worse in executive functions tests FAB (12.81 ± 3.55 vs. 13.65 ± 2.57; *F* = 5.6, *p* = 0.02) and Digit symbol (33.46 ± 18.581 vs. 39.70 ± 13.08; *p* = 0.005) and showed more depressive symptoms SCID (1.46 ± 2.70 vs. 0.67 ± 1.94; *p* = 0.014) than the robust group. But there were neither significant differences among frailty categories in the global cognition test MMSE (27.82 ± 1.48 vs. 28.08 ± 1.20; *p* = 0.676) nor in the episodic memory tests, measured by the FCSRT (42.95 ± 10.57 vs. 43.34 ± 0.07; *p* = 0.751) (Table 1).

**TABLE 1:** Comparisonbetween frailty phenotype groups.

Variable	Robust			Pre-frail and Frail			*p*
**N/%**	**74/65.5%**			**39/34.5%**			
Sex (male/female)	33/41			10/29			0.066
	**Mean**	**Median**	**Min/Max**	**Mean**	**Median**	**Min/Max**	***p***
Years of age	73.2 ± 5.54	72	65/86	75.08 ± 5.51	75	65/88	0.097
Years of education	10 ± 4	12	0/20	8 ± 4	8	1/20	0.051
**VA right**	**45.02 ± 31.44**	**40**	**20/100**	**55.64 ± 43.06**	**50**	**25/200**	**0.039**
VA left	43.06 ± 30.62	40	20/200	50.92 ± 44.57	50	25/200	0.129
PTA right	28.42 ± 14.20	25	10/53	30.68 ± 19.72	30	6/70	0.330
PTA left	27.18 ± 14.45	26	10/51	29.54 ± 20.10	26	8/74	0.321
**Charlson comorbidity index**	**0.67 ± 1.46**	**0**	**0/4**	**1.24 ± 2.04**	**1**	**0/6**	**0.020**
**Timed up and go (sec)**	**8.27 ± 3.53**	**8**	**3/20**	**9.49 ± 4.92**	**9**	**6/29**	**0.037**
Hypertension%	66.2			74.4			0.399
Diabetes mellitus%	29.7			25.6			0.826
Smoking%	23			20.5			0.816
CDR SOB	0.25 ± 0.64	0	0/2	0.31 ± 0.90	0	0/2.5	0.562
**Frontal assessment battery**	**13.65 ± 2.57**	**14**	**10/18**	**12.81 ± 3.55**	**12.81**	**8/18**	**0.047**
**Digit symbol**	**39.70 ± 13.08**	**39**	**14/69**	**33.46 ± 18.58**	**28**	**5/63**	**0.005**
FCSRT total score	43.34 ± 8.07	45	21/48	42.95 ± 10.57	45	8/48	0.751
MMSE	28.08 ± 1.20	28	24/30	27.82 ± 1.48	28	22/30	0.676
**Total SCID**	**0.67 ± 1.94**	**0**	**0/6**	**1.46 ± 2.70**	**0**	**0/8**	**0.014**

VA, visual acuity; PTA, pure tonal average; CDR SOB, clinical dementia rating sum of boxes; Digit Symbol, digit symbol substitution test; FCSRT, free and cued selective reminding test; MMSE, mini-mental state examination, SCID, structured clinical interview for depression for DSM disorders. Significant differences between groups (*p* < 0.05) are bolded.

When comparing the sum of the frontal WMH score between groups, the frail/pre-frail group (median, [50 CI]) = 2 [2, 4]) had significant larger F-WMH (*U* = 1005.5, *z* = −2.57, *p* = 0.01) than the robust one (2, [1, 2]).

### 3.3. Correlations between executive functions, frailty sore, and frontal WMH

**FIGURE 1:**
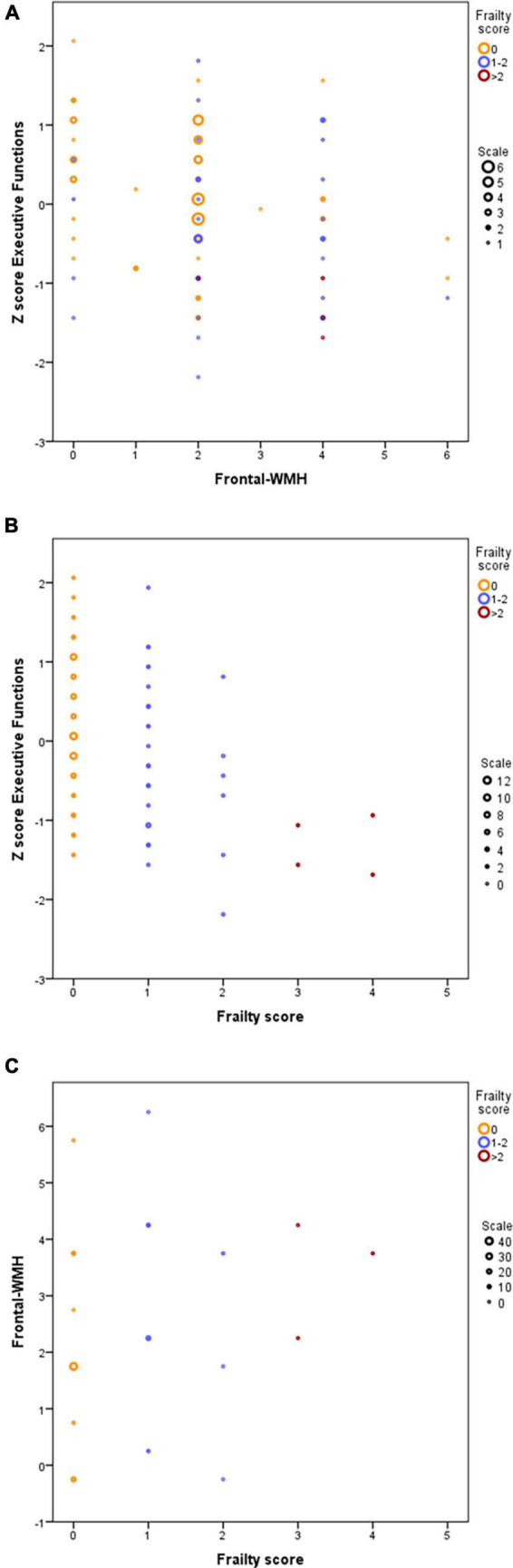
Correlations between frailty score. F-WMH and executive functions. **(A)** Z-scores of executive functions are inversely correlated with frontal WMH scores. **(B)** Z-scores of executive functions are inversely correlated with frailty scores. **(C)** Frontal WMH are directly correlated with frailty scores. Symbol sizes in bubble plots represent the number of individuals for each (X, Y) data point: yellow means robust (frailty score = 0), blue means prefrail (1–2) and red means frail (>2). 0: robust individuals; 1–2: Pre-frail individuals; >2: frail individuals.

Executive function composite Z score was inversely correlated with frontal-WMH (*r* = −0.244, *p* = 0.01); also, executive functions were inversely correlated with frailty scores (*r* = - 0.35, *p* < 0.001); Finally, frontal-WMH was directly correlated with physical frailty scores (*r* = 0.264, *p* = 0.005) (Figures 1A–C).

### 3.4. Mediation model

**FIGURE 2:**
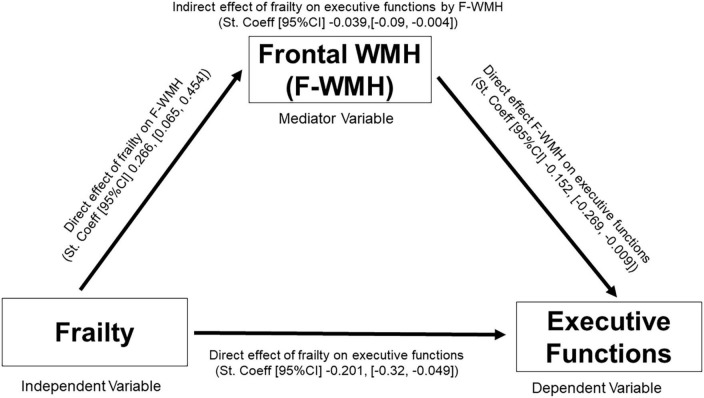
Mediation scheme. The objective of this study is to assess if WMH mediate the association between frailty and executive functions in older adults without dementia. F-WMH, frontal white matter hyperintensities, St Coeff., standardized coefficient; CI, confidence interval.

Firstly, we analyzed if frailty scores, age, sex and education could explain the variance of frontal-WMH (model 1) and found that the model explained 11.21% of the variability in frontal-WMH, with a significant effect of frailty (Standardized Coeff, [95% CI] −0.266 [0.065, 0.454]) and without significant effects for the other variables. In the second regression (model 2) we analyzed the effects of the frailty score, F-WMH, age, sex and education on executive functions, observing that this model explained 52.32% of the variability in executive functions. There was a significant effect of frailty (Standardized Coeff, [95% CI]) = −0.201, [−0.319, −0.049], F-WMH (−0.152, [−0.269, −0.009]), age (−0.319, [−0.423, −0.162]), sex (0.209, [0.134, 0.651]), and education (0.435, [0.268, 0.519]). Finally, a third model (model 3) was calculated analyzing the effects of the frailty score, age, sex and education on executive functions, this model explained 50.27% of the variability in executive functions, showing significant effects of frailty (−0.240, [−0.354, −0.087]), age (−0.345, [−0.446, −0.185]), sex (0.204, [0.119, 0.645]), and education (0.422, [0.254, 0.509]) on executive functions. With this analysis the mediation model revealed that frontal-WMH (−0.039, [−0.09, −0.004]) partially mediate the relation between frailty score and executive functions (Figure 2 and Table 2).

**TABLE 2:** Mediation model.

	Standardized Coeff, [95% CI]	Coeff.	SE	*t*	*p*	LLCI	ULCI
**Model 1 summary *R* = 0.3348, *R*-sq = 0.1121, *F* = 3.3136, *p* = 0.0134 *Y* = F-WMH, *X* = frailty score. Covariables: age, educational level, sex.**							
Constant		−1.7793	1.9554	−9099	0.3649	−5.6565	2.0979
Frail score	0.266, [0.065, 0.454]	0.4665	0.1763	2.6470	0.0094	0.1171	0.8160
Age	0.170, [−0.020, 0.360]	0.0458	0.0259	1.7702	0.0796	−0.0055	0.0970
Education	0.090, [−0.097, 0.275]	0.0311	0.0327	0.9502	0.3442	−0.0338	0.0959
Sex	0.037, [−0.308, 0.458]	0.1114	0.2864	0.3890	0.6980	−0.4564	0.6792
**Model 2 summary *R* = 0.7233, *R*-sq = 0.5232, *F* = 22.8264, *p* < 0.0001 *Y* = Z score executive functions, *X* = frailty score; F-WMH, Covariables: age, educational level, sex**							
Constant		3.1068	0.8935	3.4773	0.007	1.3350	4.8786
Frailty score	−0.201, [−0.319, −0.049]	−0.2230	0.0829	−2.6920	0.0083	−0.3873	−0.0587
F-WMH	−0.152, [−0.269, −0.009]	−0.0939	0.0444	−2.1147	0.0368	−0.1820	−0.0058
Age	−0.319, [−0.423, −0.162]	−0.0531	0.0119	−4.4450	<0.0001	−0.0768	−0.0294
Education	0.435, [0.268, 0.519]	0.0926	0.0149	6.1934	<0.0001	0.0629	0.1222
Sex	0.209, [0.134, 0.651]	0.3925	0.1304	3.0096	0.0033	0.1339	0.6512
**Model 3 summary *R* = 0.7090. *R*-sq = 0.5027. *F* = 26.5375. *p* < 0.0001 *Y* = Z score executive functions, *X* = frailty score; Covariables: age. educational level. sex**							
Constant		3.2740	0.9046	3.6194	0.005	1.4804	5.0675
Frailty score	−0.240, [−0.354, −0.087]	−0.2669	0.0815	−3.2730	0.0014	−0.4285	−0.1052
Age	−0.345, [−0.446, −0.185]	−0.0574	0.0120	−4.7975	<0.0001	−0.0811	−0.0337
Education	0.422, [0.254, 0.509]	0.0897	0.0151	5.9267	<0.0001	0.0597	0.1197
Sex	0.204, [0.119, 0.645]	0.3821	0.1325	2.8841	0.0048	0.1194	0.6447
**Direct effect of frailty score over executive function**							
Frailty score	−0.201, [−0.32, −0.049]	−0.2230	0.0829	−2.6920	0.0083	−0.3873	−0.0587
**Indirect effect of frailty score over executive function through F-WMH**							
F-WMH	−0.039, [−0.09, −0.004]	−0.0438	0.0221			−0.0899	−0.0032

F-WMH, frontal white matter hyperintensities; CI, confidence interval; LLCI, lower limit confidence intervals (2,5%); ULCI, upper limit confidence intervals (97,5%); Coeff, coefficient; SE, standard error.

## 4. Discussion

As far as we know, this is the first research studying the neural links of the executive dysfunction in frail syndrome. Our model showed that 52% of the variance of executive functions could be explained by the frailty phenotype, age, scholarship, sex and frontal-WMH. Further on, we found that frontal-WMH partially mediate the association between frailty and executive dysfunction, explaining a small amount of the neural links between these common geriatric syndromes.

Our results are consistent with growing evidence that shows an association between frailty, cognitive impairment, and subcortical white matter damage (Maltais et al., 2019; Siejka et al., 2020; Ducca et al., 2023). Previous studies have shown that subjects with cognitive frailty had higher volumes of subcortical WMH than subjects with physical frailty without cognitive impairment, and than subjects with mild cognitive impairment but without physical frailty (Sugimoto et al., 2019; Yoshiura et al., 2022). Despite this associations F-WMH only explain a very small amount of the association (Standardized Coeff. = −0.0395) between frailty and executive dysfunction.

In order to explain the small mediation effect of the F-WMH in the association between frailty and executive dysfunction it is important to highlight that there are many other neuropathological changes related with frailty that could also mediate this association, including other indicators of vascular disease, neurodegenerative diseases and subcortical brain atrophy, which were not measured in our study. In fact, frailty has been associated with neuropathological brain changes of many neurodegenerative diseases: Alzheimer’s Disease, Lewy Body Disease, and nigral neuronal loss pathology (Buchman et al., 2013). In addition to the WMH, cognitive frailty has been associated with other indicators of small vessel disease, such as microbleeds and lacunar infarcts (Yoshiura et al., 2022). Also, it has been showed that cognitive frailty is associated with significant volume reductions in various subcortical nuclei: bilateral thalami, left caudate, right pallidum, and accumbens area (Wan et al., 2020) as well as with atrophy in the hippocampus, amygdala, parahippocampal gyrus, and entorhinal cortex (Yoshiura et al., 2022).

Other possible explanations for the association of frailty and executive dysfunction could be associated with neurofunctional changes, it have been described an hypothalamic–pituitary axis dysfunction in frail subjects (Clegg and Hassan-Smith, 2018). Similarly, chronic inflammation has been described in frail subjects, with elevated concentrations of IL-6, TNF-a, and C reactive protein (Alberro et al., 2021) as well as higher level of fibrinogen and white blood cells count, compared with robust individuals (Soysal et al., 2016). Interestingly, several studies have postulated the existence of abnormally elevated levels of inflammatory cytokines in subjects with affective disorders (Serafini et al., 2020) which may explain the relationship between frailty and neuropsychiatric symptoms, that in our study was expressed by higher depressive symptoms in the frail/pre-frail group.

Regarding the subcortical-frontal cognitive profile found in frail/pre-frail subjects of our study, previous studies have identified similar profiles, with associations between frailty and executive dysfunction but not with memory domains (Delrieu et al., 2016), confirming the subcortical frontal aspects of the frailty syndrome. Because we did a cross sectional analysis it is not possible to stablish a directionality in the association between frailty and executive dysfunction. In fact, the effects of childhood educational levels and age were larger than the effects of frailty in executive functions. Furthermore, executive function impairment on its own could be a predictor of frailty and disability in older adults, as it was observed in a study by Gross et al. (2016) that followed 331 women free from dementia and frailty, showing that impaired executive functioning was associated with frailty onset. Indeed, this association is an example of impairment in goal-directed behaviors, where lack of initiative or planning due to executive dysfunction could promote physical inactivity and subsequent sarcopenia associated with frailty (Hollocks et al., 2015).

Our results are especially relevant for prevention in older adults, because we have shown that even in the early stages of physical frailty (pre frail subjects) there is an association with executive dysfunction and increased frontal-WMH (Ruan et al., 2015). Early intervention of cardiovascular risk factors across life span (Afilalo et al., 2009) and promoting healthy aging with multiple interventions in lifestyle, like increasing physical activity may reduce vascular burden and therefore reduce the risk of frailty and cognitive impairment (Ngandu et al., 2015). Nutritional changes are recommended for frail subjects, since some essential amino acids have demonstrated to be decreased in subjects with malnutrition (Aquilani et al., 2020).

Our study has several limitations: 1. Only relatively healthy adults were included in the Andes cohort, because the inclusion and exclusion criteria leave out subjects with stroke, other neurological disorders, and prior psychiatric disorders, reducing the chances of finding significant cerebrovascular disease, cognitive impaired and frail subjects. In fact, in our cohort only 4% of the patients were categorized as frail, which is much lower compared to the prevalence of 13.9% reported in the Chilean community dwelling population older than 60 years (Leiva et al., 2020). 2.-We did not measure other types of brain vascular lesions (like lacunes or microbleeds) in our analysis, which had been related with cognitive frailty. 3.-Because we did a cross sectional analysis, no causation could be concluded from the associations found. 4.-Finally we have a quite small sample size, augmenting the possibilities of type 2 errors.

In conclusion, our results suggest that even at early stages of physical frailty, there is a significant association between frailty and cognitive impairment expressed as executive dysfunction, and brain frontal-WMH, revealing the importance of preventing these common geriatric syndromes. Frontal-WMH are directly related with executive dysfunction, but also, they partially mediated the association between frailty and executive dysfunction. Many other causes of brain changes associated with frailty may also explain the association between frailty and executive dysfunction, further larger longitudinal studies that include multiple neuroimaging biomarkers are needed to analyze in detail the neural links of cognitive dysfunction in frailty.

## Data availability statement

The raw data supporting the conclusions of this article will be made available by the authors, without undue reservation.

## Ethics statement

The studies involving human participants were reviewed and approved by the Comite Cientifico Hospital Clínico Universidad de Chile. The patients/participants provided their written informed consent to participate in this study.

## Author contributions

CD: study conception and design. CD, NP, CR, PD, MT, MA, MR, CF, and PO: data collection. CD, NP, PD, CR, and VM: analysis and interpretation of results. CD, NP, CR, PD, VM, and MA: draft manuscript preparation. CD, PD, PO, VM, MR, CF, MT, and MA: revision of the manuscript. All authors reviewed the results and approved the final version of the manuscript.
